# Commentary: Causal relationship between particulate matter 2.5 and diabetes: two sample Mendelian randomization

**DOI:** 10.3389/fpubh.2024.1353982

**Published:** 2024-02-26

**Authors:** Yao Ni, Youqian Zhang, Jianzhou Ye, Xuesong Yang

**Affiliations:** ^1^Department of Dermatovenereology, Chengdu Second People's Hospital, Chengdu, Sichuan, China; ^2^Health Science Center, Yangtze University, Jingzhou, Hubei, China; ^3^Department of Dermatology, Yunnan Provincial Hospital of Traditional Chinese Medicine, The First Affiliated Hospital of Yunnan University of Chinese Medicine, Kunming, Yunnan, China

**Keywords:** Mendelian randomization, particulate matter 2.5, diabetes, environmental epidemiology, genome-wide association study (GWAS)

Diabetes mellitus (DM) is a significant metabolic disorder characterized by chronic hyperglycemia and is showing an increasing trend globally (1). Recent research has indicated that particulate matter 2.5 (PM_2.5_) can induce a pro-inflammatory state, increasing oxidative stress, thereby elevating the risk of DM, a condition detectable through the measurement of homocysteine levels (2–4). However, due to the inherent limitations of traditional research methodologies, the causal relationship remains unconfirmed. Mendelian randomization (MR) uses single nucleotide polymorphisms (SNPs) from genome-wide association studies (GWASs) as instrumental variables (IVs) to assess the impact of exposure on outcomes. This method can avoid the influence of confounding factors and reverse results (5). Consequently, we were intrigued by the recent study conducted by Kim et al., which employed the two-sample MR (TSMR) approach in investigating the causal relationship between PM_2.5_ and DM (6).

Kim et al.'s TSMR analysis primarily indicated that genetic susceptibility to PM_2.5_ is associated with a higher risk of DM. However, the dataset used for the analysis had significant shortcomings, leading to potential false-positive results and considerable bias in their findings. Specifically, the DM dataset originated from the UK Biobank (UKB), and notably, the exposure dataset was also sourced from UKB. This contravenes the principles of TSMR studies based on summary-level GWAS data, with an astonishingly high sample overlap rate of 91.81% calculated. Additionally, since Type 1 DM (T1DM) is an autoimmune disease and existing observational studies primarily focus on PM_2.5_ and Type 2 DM (T2DM) (4), the MR study's use of generalized DM data is problematic. The UKB describes DM as “Has a doctor ever told you that you have diabetes?” which lacks specificity in diabetes typing. Furthermore, according to the publication date of the article, the most up-to-date and comprehensive GWAS data should be used to ensure scientific advancement, a detail the authors overlooked. We reanalyzed the data using Kim et al.'s method and other approaches, differentiating DM types. We obtained the most current and comprehensive data on European ancestry T2DM (80,154 cases/853,816 controls) from the DIAbetes Genetics Replication And Meta-analysis (DIAGRAM) consortium (7), and T1DM data (4,196 cases/308,252 controls) from the FinnGen consortium (8). Sensitivity analyses were conducted which included additional calculations of I^2^ to assess the suitability of fixed effects in the inverse-variance weighted (IVW) method (Table 1). Following the application of the Bonferroni correction (*P* < 0.05/2), our MR analysis validated a significant causal association between PM_2.5_ and T2DM (Figure 1). Discrepancies in the outcomes can be attributed to the inadequate consideration of the DM phenotype and misunderstanding of the TSMR principle in the initial MR study conducted by Joyce Mary Kim et al., which subsequently resulted in false-positive findings. We acknowledge the challenges often faced when analyzing risk factors such as PM_2.5_ and appreciate the authors' endeavors in advancing our comprehension of the intricate connection between PM_2.5_ and DM. However, in this intricate field, precise identification of datasets and adherence to the TSMR principle are imperative in order to substantiate conclusions regarding potential causality between clinical characteristics and/or diseases.

**Table 1:** Summary of sensitivity results.

**Traits**	**MR-Egger intercept**			**MR-PRESSO global test**			**Cochrane's** ***Q***				**Steiger_test**	
	**Intercept**	**SE**	***Pval***	**RSS_obs_**	***P*-value**	***Outlier***	***Q***	***Q_df***	***Q_pval***	***I*^2^ (%)**	**Direction**	***Pval***
PM_2.5_ on T1DM	0.023	0.015	0.187	14.541	*0.468*	NA	6.691	6	0.350	10	TRUE	1.07E-10
PM_2.5_ on T2DM	0.004	0.009	0.681	9.546	*0.315*	*rs77205736*	7.232	5	0.204	31	TRUE	5.91E-20

MR, Mendelian Randomization; MR-PRESSO, MR Pleiotropy Residual Sum and Outlier; PM2.5, particulate matter 2.5; T1DM, Type 1 Diabetes mellitus; T2DM, Type 2 Diabetes mellitus.

**Figure 1:**
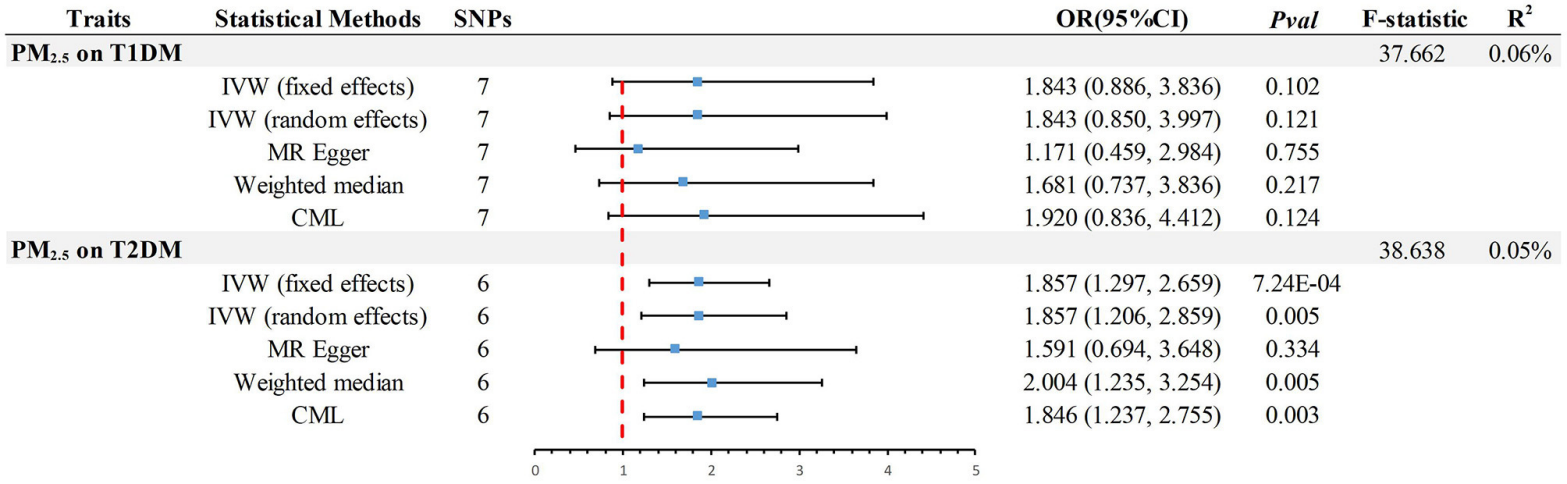
Summary of estimates of genetically predicted PM_2.5_ for causality of T1DM or T2DM as assessed by IVW, the primary method of Mendelian randomization. MR, Mendelian randomization; IVW, Inverse-Variance-Weighted; CML, Conditional Maximum likelihood; OR, odds ratio; CI, confidence interval; T2DM, Type 2 Diabetes Mellitus; T1DM, Type 1 Diabetes Mellitus; SNP, Single Nucleotide Polymorphism; PM_2.5_, particulate matter 2.5.

In the analysis of MR, the *F*-statistic plays a crucial role in evaluating the instrumental variables (IVs) and their ability to explain the exposure variable. Additionally, the variables *R*^2^ and POWER are important indicators used to measure the proportion of explained variance and the statistical test's power, respectively. In MR study, the presence of weak IVs can potentially skew the estimates and results. To mitigate this issue, researchers rely on the *F*-statistic, which should ideally exceed a value of 10 to ensure the IVs possess sufficient strength for accurate estimation of causal associations between exposure and outcome (9). Additionally, ensuring the validity and dependability of outcomes requires adequate statistical power. Insufficient power frequently leads to an incapability of precisely identifying the extent of causal consequences, amplifying the likelihood of erroneous positive findings. Hence, power analysis is of utmost significance. Considering that the majority of genetic variations account for a minute portion of phenotypic variability, statistical power is considered a prominent obstacle in MR investigations (10). However, the authors, Kim et al., overlooked these crucial factors, and the absence of such critical statistical information could potentially lead to misinterpretation of the study findings. This, in turn, may have a significant impact on the overall quality and credibility of the research conducted. To address this concern, we conducted additional calculations, including evaluating the *F*-statistic [*F* = *R*^2^ × (N - 2)/(1 - R^2^)] (9), determining the *R*^2^ value [*R*^2^ = 2 × MAF × (1 - MAF) × beta^2^, where MAF represents the minor allele frequency for each single nucleotide polymorphism] (11), and assessing statistical power (https://shiny.cnsgenomics.com/mRnd/). The results revealed that in the analysis of PM_2.5_ with T1DM and T2DM, six (Outlier:rs77205736) and seven IVs were identified (Figure 1), respectively. The average *F*-statistic for both exceeded 37, indicating robust instrument strength. The genetic variance explained by PM_2.5_ was 0.06% for T1DM and 0.05% for T2DM. Using odds ratios derived from the IVW method, the power values for detecting associations in PM_2.5_ with T1DM and T2DM were 96 and 100%, respectively. This confirms the reliability of the causality.

This commentary thoroughly investigates the intricate connections between PM_2.5_ and DM. The original study conducted by Kim et al. established a crucial groundwork for comprehending these associations through MR analysis. They posit that there exists a genetic-level causal relationship between PM_2.5_ and diabetes in the European population. However, our reevaluation, utilizing refined definitions of phenotypes and expanded analytical techniques, which encompass meticulous assessment of instrumental variables and power analysis, presents a more nuanced standpoint. Our findings suggest that PM_2.5_ lacks a noteworthy causal association with type 1 diabetes, but does elevate the risk of type 2 diabetes. In order to uphold scientific transparency, we express our gratitude to the editors of “Frontiers in Public Health” and the authors of the paper for recognizing and addressing our concerns.

## Author contributions

YN: Conceptualization, Data curation, Formal analysis, Investigation, Methodology, Resources, Supervision, Writing – original draft, Writing – review & editing. YZ: Conceptualization, Formal analysis, Investigation, Methodology, Resources, Supervision, Writing – original draft, Writing – review & editing. JY: Conceptualization, Formal analysis, Methodology, Resources, Supervision, Writing – review & editing. XY: Conceptualization, Formal analysis, Methodology, Resources, Supervision, Writing – review & editing.
